# Expanding the Hygiene Hypothesis: Early Exposure to Infectious Agents Predicts Delayed-Type Hypersensitivity to *Candida* among Children in Kilimanjaro

**DOI:** 10.1371/journal.pone.0037406

**Published:** 2012-05-17

**Authors:** Katherine Wander, Kathleen O'Connor, Bettina Shell-Duncan

**Affiliations:** 1 Department of Anthropology, University of Washington, Seattle, Washington, United States of America; 2 Center for Studies in Demography and Ecology, University of Washington, Seattle, Washington, United States of America; University of Ottawa, Canada

## Abstract

**Background:**

Multiple lines of evidence suggest that infections in early life prevent the development of pathological immune responses to allergens and autoantigens (the *hygiene hypothesis*). Early infections may also affect later immune responses to pathogen antigen.

**Methods:**

To evaluate an association between early infections and immune responses to pathogen antigen, delayed-type hypersensitivity (DTH) to *Candida albicans* was evaluated among 283 2- to 7-year-old children in Kilimanjaro, Tanzania. A questionnaire and physical examination were used to characterize variables reflecting early exposure to infectious agents (family size, house construction materials, BCG vaccination, hospitalization history). Logistic regression was used to evaluate the association between early exposure to infectious agents and DTH to *C. albicans*.

**Results:**

Triceps skinfold thickness (OR: 1.11; 95% CI: 1.01, 1.22) and age (OR: 1.27; 95% CI: 1.04, 1.55) were positively associated with DTH to *C. albicans*. Adjusted for age and sex, large family size (OR: 2.81; 95% CI: 1.04, 7.61), BCG vaccination scar (OR: 3.10; 95% CI: 1.10, 8.71), and hospitalization during infancy with an infectious disease (OR: 4.67; 95% CI: 1.00, 21.74) were positively associated with DTH to *C. albicans*.

**Conclusions:**

Early life infections were positively associated with later DTH to *C. albicans*. This result supports an expansion of the hygiene hypothesis to explain not only pathological immune responses to allergens, but also appropriate immune responses to pathogens. Immune system development may be responsive to early infections as an adaptive means to tailor reactivity to the local infectious disease ecology.

## Introduction

The *hygiene hypothesis* [1] proposes that infections during early life shape the development of the immune system, preventing subsequent development of allergic disease. Since its initial publication, a large body of epidemiological evidence has accumulated in support of the hygiene hypothesis. Exposure to other children (presumably a source of transmission of infectious agents) through larger family size [1]–[7], higher birth order [3], or day care attendance [8]–[10], is inversely associated with allergic disease; episodes of routine infection in early life [11]–[15] and seropositivity to common viruses and bacteria (including hepatitis A virus, herpes simplex virus type 1, cytomegalovirus, *Toxoplasma gondii*, and *Helicobacter pylori*) are also associated with lower risk of allergic disease [16]–[19]. Although inconsistent findings have been reported, overall evidence supports the hypothesis that early exposure to infectious agents mitigates subsequent allergic disease risk (for further review, see refs. 5–7).

The hygiene hypothesis has been expanded to explain risk of autoimmune disease, as well. Although some infections can potentiate autoimmune responses (e.g., *Streptococcus pyogenes* pharyngitis results in acute rheumatic fever in ∼3% of untreated cases [20]), in general, early life infections seem to be protective. For example, early day care attendance [21] and older siblings or larger family size [22]–[25] are inversely associated with type 1 diabetes; large family or household size are also inversely associated with multiple sclerosis [26], [27] and inflammatory bowel disease [28]–[31]; and, *H. pylori* infection is inversely associated with inflammatory bowel disease [31].

Despite the importance of immune responses to pathogens in combating infectious disease, and the clear evidence from the epidemiology of allergy and autoimmune disease that early infections impact later immune responsiveness, relatively few studies have investigated the impact of early exposure to infectious agents on later immune responses to *pathogens*. We hypothesize that immune system development responds adaptively to stimulation from infectious agents during infancy with enhanced immune responses to pathogen antigen during childhood. To test this hypothesis, we evaluated the effect of indicators of early infectious disease morbidity on delayed-type hypersensitivity (DTH) to *Candida albicans*, a ubiquitous fungal pathogen, among children in Kilimanjaro, Tanzania, a population subject to high infectious disease stress.

Delayed-type hypersensitivity testing introduces a small amount of pathogen antigen intradermally (under the most superficial layers of the skin). Local activation of an immune response by memory T cells results in the formation of induration and erythema at the site of the injection. *C. albicans* is the causative agent of oral candidiasis (“thrush”) and vaginal candidiasis (“yeast infection”); among profoundly immunocompromised individuals, *C. albicans* can cause candidemia, a potentially fatal systemic infection. Most individuals can be assumed to have formed immunological memory of *C. albicans*; immunocompetent individuals mount a cell-mediated DTH response upon skin testing with *C. albicans*, visible as an induration at the test site. Thus, DTH to the recall antigen *C. albicans* provides a means to evaluate cell-mediated reactivity to pathogen antigen. We evaluated family size and other indicators of elevated exposure to infectious agents during infancy as predictors of DTH to *C. albicans*.

## Materials and Methods

### Participants

This project was carried out in 2 adjoining villages in the Machame area of Kilimanjaro, Tanzania (the study villages are largely similar and children were not expected to differ in any systematic way). Infectious disease is quite common among children in Kilimanjaro, including viral, bacterial, protozoan, fungal, and helminth infectious agents. Children are exposed to infectious agents through multiple aspects of life in Kilimanjaro; some examples include bed-sharing with siblings and other children, interaction with household animals, contact with contaminated soil and water (rivers and irrigation ditches), and contact with insect vectors. Piped, treated drinking water was not available in the study area at the time of data collection. 314 2–7 year old children participated in the project, randomly sampled from a census of all 2–7 year old children in the study area. Children were invited to participate if they were living with at least 1 parent and had been living in the study area for at least 6 months.

*Data collection:* Data collection was conducted over the course of 4 weeks in spring of 2010. Children and their primary caregiving parents participated in 2 days of data collection. Data were collected at a healthcare facility belonging to Nshara Community Medical Centre (NCMC). Data collection was conducted by the lead author and 4 field assistants. Field assistants were residents of the study area and medical personnel (2 nurse/midwives, 1 nurse's assistant, 1 physician) who were trained in data collection techniques prior to data collection. Written informed consent was obtained from parents of all participating children. Procedures and data collection protocols were approved by the Institutional Review Board of the University of Washington and the Tanzanian National Institute for Medical Research (NIMR) and research permission obtained from the Tanzania Commission for Science and Technology (COSTECH).

On the first data collection visit, participating children's primary caregivers completed a questionnaire (in Swahili), providing household and family demographic information, as well as information about the child's medical history. Each child's finger was pricked with a sterile lancet to obtain capillary whole blood; blood was immediately tested for HIV (SD BioLine HIV-1/2 3.0 rapid HIV-1/2 test) and hemoglobin concentration (Hb; HemoCue Hb 201^+^ hemoglobin system). Additional blood was dropped on filter paper (Whatman #903 Protein Saver Cards) and stored as dried blood spots (DBS). The Candin (Allermed Laboratories, Inc., San Diego, CA) skin test for DTH to *C. albicans* antigen was administered intradermally on each child's forearm by injecting 0.1 mL under the most superficial layers of the skin with a 27 gauge tuberculin syringe. Children experiencing symptoms of infectious disease, HIV positive children, and anemic children were referred to NCMC for additional care.

On the second data collection visit, ∼24 h after the first, the site of the Candin skin test was evaluated for the presence of an induration; the size of any induration measured in millimeters (mm) across 2 perpendicular diameters. The presence of a bacille Calmette-Guérin (BCG) vaccination scar on the child's shoulder was recorded. Weight was measured with a digital scale and height measured with an anthropometer. Mid-upper arm circumference (MUAC) was measured using a body tape. Triceps skinfold (TSF) thickness was measured using a Lange Skinfold Measurement Caliper (Graham-Field). All anthropometric measurements and Candin skin test evaluations were performed by the lead author without knowledge of the child's interview data to maintain measurement consistency and avoid bias.

### Laboratory analysis

DBS were allowed to dry at room temperature for <24 hours, and were then frozen until shipped on dry ice to the University of Washington, where they were frozen until assay (∼4 months). DBS were analyzed in the Biological Anthropology and Biodemography Laboratory at the UW for C-reactive protein (CRP) and α_1_-acid glycoprotein (AGP, or orosomucoid), 2 acute phase reactants and indicators of infection. CRP was assessed with an in-house DBS assay described elsewhere [32]. AGP was assessed with a commercially available kit (GenWay Biotech), modified for use with DBS samples.

### Data analysis

The outcome of interest, DTH to *C. albicans*, was defined as an induration size ≥5 mm in mean diameter (and anergy to *C. albicans* as induration <5 mm). We identified 4 indicators of early exposure to infectious agents (predictors of interest): *Family size* was characterized as small (≤3 cohabiting siblings) or large (>3 cohabiting siblings) based on the number of siblings living in the household at the time of participation in the project. *House construction materials* were categorized as earth or cement based on parents' report. *Hospitalization with an infectious disease during infancy* (before the first birthday) was assessed based on parents' report of the timing and diagnosis of any hospitalizations in the child's medical history. *BCG* was considered present if a BCG vaccination scar was observed during physical examination. Potential control variables were defined as follows: Age was calculated from the child's reported date of birth. Anthropometric measurements were used to define categorical variables for malnutrition. Z-scores were calculated (EPI INFO; Centers for Disease Control and Prevention, Atlanta, GA) for weight for height (WHZ), weight for age (WAZ), and height for age (HAZ). WHZ<−2 defined wasting and HAZ<−2 defined stunting. Elevated CRP and AGP values were used to identify acute infection [33]. Breastfeeding and age at weaning were described based on parents' report. Anemia was defined based on Hb values following WHO guidelines [34].

Data analysis was performed using Stata 11.2 software (Statacorp; College Station, TX). Logistic regression was used to test the predicted relationship between early life infectious disease variables and DTH. Significance was defined as p≤0.05. Age and sex were included in all models; additional control variables were retained in the model of early life predictors of DTH to *C. albicans* if their inclusion substantially altered the estimated effect of a predictor of interest (>20% change in odds ratio; this threshold was chosen to allow control for substantial confounding while preserving statistical power by limiting the number of variables included in the model).

## Results

Complete information (excluding anthropometry and biomarker analysis) was available for 283 children (age was unavailable for 17 children; information regarding siblings living in the household was unavailable for 2 children; information regarding BCG vaccination scar was missing for 2 children; and data from 10 children were excluded from analysis: 1 was HIV positive; 9 exhibited Candin skin test administration problems). Complete information including biomarkers of inflammation was available for 264 children; complete information including anthropometry was available for 240 children (43 children refused TSF measurement). Children from the 2 study villages were largely similar: significant differences were observed in only 2 variables (HAZ and house construction materials). As such, data from villages were pooled for all analyses.

Sample characteristics are described in Table 1. 54.42% of children were DTH positive to *C. albicans*, indicating cell-mediated immunocompetence. DTH to *C. albicans* did not substantially differ among the children for whom age was unknown (52.94% DTH positive) or TSF was not measured (46.51% DTH positive). Just over half (53.00%) of the sample was female.

**Table 1:** Sample characteristics.

Sex	Female	150	53.00%
	Male	133	47.00%
Age in years, mean (SD)		4.50	(1.65)
House materials	Cement	145	51.24%
	Earth	138	48.76%
Coffee farming household		110	38.87%
Anthropometry	Wasting^a^	1	0.37%
	Stunting^b^	74	27.72%
	Triceps skinfold in mm, mean (SD)	11.73	(3.24)
Siblings living in the household, mean (range)		1.71	(0, 6)
Large family (>3 siblings)		23	8.13%
BCG vaccination scar present		264	93.29%
Hospitalization in first year of life		17	6.01%
Hospitalization in first year of life for infectious disease^c^		13	4.59%
Fever (at the time of data collection)		7	2.47%
Elevated AGP or CRP^d^ (at the time of data collection)		150	57.25%
Currently breastfeeding		21	7.42%
Mean age at weaning in years (SD)^e^		1.90	(0.74)
Anemic (at the time of data collection)^f^		89	31.56%
DTH to *Candida albicans* g		154	54.42%

a Weight for height Z-score<−2; information available for 270 children.

b Height for age Z-score<−2; information available for 270 children.

c Parents' report of physician's diagnosis of pneumonia, malaria, or diarrhea for hospitalization before 1 year of age.

d C-reactive protein (CRP) >1.1 mg/L or α_1_-acid glycoprotein (AGP) >0.76 g/L, indicative of acute infection [33]; information available for 264 children.

e Among those children who are not currently breastfeeding.

f Information available for 282 children.

g Candin skin test; induration ≥5 mm in diameter indicates positive delayed-type hypersensitivity (DTH).

Family size ranged from 0 to 6 siblings (in addition to the participating child), with 8.13% of children living in “large” families (>3 siblings). Just under half (48.76%) of participating children lived in houses constructed from earth. Most (93.29%) exhibited a discernible BCG vaccination scar. For those children hospitalized in the first year of life (17), 13 (4.59% of participating children) reported an infectious disease diagnosis (pneumonia, 10; malaria, 2; diarrhea, 1); the other 4 reported diagnoses were ambiguous or non-infectious in nature (chest tightness, stomachache, intussusception, and jaundice).

Logistic regression results for known risk factors for immunosuppression and DTH to *C. albicans* are shown in Table 2. TSF (a measure of adiposity that is reduced in children undergoing protein energy malnutrition) and age were both positively associated with DTH to *C. albicans*. Infection, as indicated by elevated acute phase reactants (CRP and AGP), fever (data not shown), or reported recent symptoms (data not shown) was not a significant predictor of DTH to *C. albicans*; use of alternative biomarker definitions of acute infection did not alter these results.

**Table 2:** Predictors of DTH to *Candida albicans*.

Variable	OR	95% CI	p-value
Male sex	1.29	0.74, 2.24	0.369
Age in years	1.27	1.04, 1.55	0.017
Triceps skinfold thickness in mm	1.11	1.01, 1.22	0.029
Elevated CRP or AGP^a^	0.87	0.51, 1.50	0.623

a C-reactive protein (CRP) >1.1 mg/L or α_1_-acid glycoprotein (AGP) >0.76 g/L, indicative of acute infection [33].

Logistic regression results for early life factors and DTH to *C. albicans* are show in Table 3. Adjusted for age and sex, large family size, BCG vaccination scar, and hospitalization in infancy with an infectious disease diagnosis were significantly positively associated with DTH to *C. albicans*. Large family size was associated with 2.8-fold higher odds of DTH to *C. albicans*; a BCG vaccination scar with 3.1-fold higher odds of DTH to *C. albicans*; and, hospitalization with an infectious disease diagnosis during infancy with 4.7-fold higher odds of DTH to *C. albicans*. House construction materials were not a significant predictor of DTH to *C. albicans* (data not shown). When family size was defined by the number of other children, rather than siblings, living in the household, results were similar (OR for >3 other children: 2.13; 95% CI: 0.99, 4.58). Consideration in separate models, adjusted for age and sex, did not substantially alter the magnitude or significance of these associations (although the association between family size and DTH was of marginal significance; data not shown), nor did additional adjustment for on-going acute infection (elevated CRP or AGP), TSF, HAZ, WHZ, breastfeeding/age at weaning, or anemia (data not shown).

**Table 3:** Early life predictors of DTH to *Candida albicans*.

Variable	OR	95% CI	p-value
Male sex	1.03	0.64, 1.67	0.899
Age in years	1.11	0.96, 1.29	0.155
Large family size (>3 siblings living in the home)	2.81	1.04, 7.61	0.041
BCG vaccination scar	3.10	1.10, 8.71	0.032
Hospitalization during infancy with an infectious disease^a^	4.67	1.00, 21.74	0.050

a Pneumonia, malaria, or diarrhea, according to parents' report.

## Discussion

We document positive associations between 3 factors reflecting early life exposure to infectious agents—family size, BCG vaccination scar, and reported hospitalization with an infectious disease diagnosis in infancy—and DTH to *C. albicans*, an indicator of cell-mediated immunocompetence.

Just over half (54.42%) of participating children exhibited DTH to *C. albicans*; this is comparable to observations among children in other East African populations [35]. To verify DTH to *C. albicans* as an indicator of cell-mediated immunocompetence, we assessed its association with known risk factors for suppressed cell-mediated immunity: male sex [36], [37], young age [38], protein-energy malnutrition [39]–[41], and acute infection [41]. DTH to *C. albicans* did not differ significantly by sex, potentially due to the young age of the participating children (excess male infectious disease morbidity and mortality may not begin until adolescence [42]). As expected, age and adiposity (TSF) were positively associated with DTH. Acute infection was not associated with DTH. With the exception of acute infection, these results are consistent with expectations for a marker of cell-mediated immunity and support the interpretation of DTH to *C. albicans* as an indicator of cell-mediated immunocompetence.

Delayed-type hypersensitivity to *C. albicans* is dependent on both immunological memory of *C. albicans* and intact cell-mediated immunity. In employing the Candin skin test, we assume that *C. albicans* is ubiquitous in the environment and that virtually all subjects have had the opportunity to form immunological memory of *C. albicans* antigen. If this assumption is faulty, it is possible that significant predictors of DTH to *C. albicans* reflect variability in exposure to *C. albicans*, rather than variability in immunocompetence. We are confident in our interpretation of DTH to *C. albicans* as a marker of immunocompetence for 2 reasons. First, it varies with known predictors of cell-mediated immunity (age and adiposity). Second, it is unlikely that the common factor between the predictors of interest (large family size, BCG vaccination scar, and history of hospitalization with an infection during infancy) is enhanced exposure to *C. albicans*. Exposure to infectious agents in early life, with lasting effects on later cell-mediated DTH, is a more likely explanation for the independent effects of these 3 variables.

In keeping with the hygiene hypothesis [1]–[7], [22]–[31], we interpret family size as an indirect indication of the infectious disease experience of early childhood: siblings are likely an important source of infectious disease transmission, and those from larger families likely experienced more frequent infections. Thus, the association between large family size and DTH to *C. albicans* can be interpreted as a positive effect of early life infections on later cell-mediated immunocompetence. We recognize limitations in family size as a predictor variable. To obtain accurate information about family size, we inquired about household composition at the time of the study, and categorized family size based on *currently* cohabiting siblings. However, it is the presence of other children in the household *during infancy* that is of interest. Household composition at the time of data collection is unlikely to perfectly reflect household composition during the period of interest, as household composition in this society can be fluid, with children and adults moving between households for a variety of reasons. We chose to base the family size variable on currently cohabiting siblings (rather than all currently cohabiting children) as a means to count only those children most likely to have been a part of the household during the participating child's infancy. The family size variable, although subject to the limitations discussed here, also has an important advantage: it allowed us to demonstrate a parallel between the epidemiology of immune-mediated disease (inversely associated with siblings [1]–[7], [22]–[31]) and DTH to *C. albicans* (positively associated with siblings).

Bacille Calmette-Guérin vaccination scars and reported hospitalizations we interpret as 2 direct measures of exposure to infectious agents. BCG vaccine is usually administered in the first week of life and exposes infants to an attenuated strain of *Mycobacterium tuberculosis*. We use BCG vaccination scar, rather than parents' report or medical records (maternal-child health clinic cards, which were often unavailable), in hopes of most accurately distinguishing those few children who were not exposed to BCG as infants. The positive association between BCG vaccination scar and DTH may reflect an effect of BCG vaccination on immune system development, or may reflect immune characteristics already in place during infancy (i.e., malnourished newborns or those with a genetic predisposition to anergy may fail to form a scar after BCG vaccination *and* may fail to exhibit DTH to *C. albicans* at age 2–7 years). This limits interpretation of the positive association between BCG scar and DTH we observed. However, such confounding does not explain the other early life predictors of DTH to *C. albicans* we report. Indeed, such an explanation would predict an *inverse* association between hospitalization and DTH, rather than the positive association we observed, as children born with a predisposition to anergy would be more susceptible to severe infectious disease during infancy.

Although parents' report of many direct measures of early exposure to infectious agents may be unreliable (e.g., routine infectious disease episodes, vaccinations), we chose to inquire into hospitalizations during the first year of life, as these were likely to be more serious, and therefore memorable, events for parents. Relying on parents' memory of the hospitalization and accurate reporting of the physician's diagnosis may well introduce classification error in both the hospitalization timing and diagnosis; however, any misclassification of hospitalizations due to inaccurate reporting by parents is unlikely to be biased with respect to DTH to *C. albicans*, and thus was unlikely to unduly affect our results.

In summary, by both indirect and direct measures, we demonstrate that exposure to infectious agents in early life is positively associated with DTH to *C. albicans* (indicative of intact cell-mediated immunity) during childhood. This result is consistent with findings from allergy and autoimmune disease epidemiology suggesting that early infections influence immune-mediated disease risk. Our results suggest expansion of the hygiene hypothesis beyond allergy and autoimmune disease: In addition to inhibiting pathological immune responses to allergens and autoantigens, stimulation by early infections may promote cell-mediated immune responses to pathogen antigen. This effect may represent an adaptive mechanism in immune system development—“priming” by exposure to infectious agents during infancy may enhance cell-mediated immune responses to infectious agents encountered later in childhood, mitigating infectious disease risk.

Our data cannot directly evaluate the mechanisms that may underlie this priming; however, our results are consistent with the hypothesis that early exposure to infectious agents stimulates deviation toward T-helper type 1 (Th1)-mediated responses (including protective responses against viral, bacterial, and protozoan infectious agents and many pathological responses against autoantigens) and away from T-helper type 2 (Th2)-mediated responses (including protective responses against helminthic agents and pathological responses against allergens) [43]. DTH responses are largely, but not exclusively, dependent on the Th1 mediator interferon-γ, and thus reflect Th1-mediated reactivity to pathogen antigen [44]. Early stimulation from viral, bacterial, and protozoan agents may stimulate deviation toward stronger Th1 responses (increasing DTH reactivity to *Candida*) and away from Th2 responses (decreasing risk of allergy). However, recent evidence suggests that priming of regulatory mechanisms that act against both Th1- and Th2-mediated responses may underlie the inverse association between early exposure to infectious agents and allergy (as well as autoimmune disease) [45], [46]. As such, early exposure to infectious agents may impact immune-mediated disease risk and DTH via distinct mechanisms. It is likely that infectious agents interact with the developing immune system in multiple and complex ways; this area is ripe for additional research.
